# Enhanced recognition memory in grapheme-color synaesthesia for different categories of visual stimuli

**DOI:** 10.3389/fpsyg.2013.00762

**Published:** 2013-10-24

**Authors:** Jamie Ward, Peter Hovard, Alicia Jones, Nicolas Rothen

**Affiliations:** Sackler Centre for Consciousness Science and School of Psychology, University of Sussex, Brighton, UK

**Keywords:** synaesthesia/synesthesia, recognition memory, scenes, color

## Abstract

Memory has been shown to be enhanced in grapheme-color synaesthesia, and this enhancement extends to certain visual stimuli (that don't induce synaesthesia) as well as stimuli comprised of graphemes (which do). Previous studies have used a variety of testing procedures to assess memory in synaesthesia (e.g., free recall, recognition, associative learning) making it hard to know the extent to which memory benefits are attributable to the stimulus properties themselves, the testing method, participant strategies, or some combination of these factors. In the first experiment, we use the same testing procedure (recognition memory) for a variety of stimuli (written words, non-words, scenes, and fractals) and also check which memorization strategies were used. We demonstrate that grapheme-color synaesthetes show enhanced memory across all these stimuli, but this is not found for a non-visual type of synaesthesia (lexical-gustatory). In the second experiment, the memory advantage for scenes is explored further by manipulating the properties of the old and new images (changing color, orientation, or object presence). Again, grapheme-color synaesthetes show a memory advantage for scenes across all manipulations. Although recognition memory is generally enhanced in this study, the largest effects were found for abstract visual images (fractals) and scenes for which color can be used to discriminate old/new status.

## Introduction

For people with grapheme-color synaesthesia, stimuli consisting of letters (including words) or digits are associated with experiences of color (Simner et al., 2006). These synaesthetes also show better memory for words, for instance in tests of free recall of lists (e.g., Radvansky et al., 2011). The most intuitive explanation for this is that it is the “extra” colors themselves that enable grapheme-color synaesthetes to create richer, and more robust, memory representations for words. In the case of spoken words, synaesthetes may encode these as visual objects enabling them to store them both as a verbal code and a visual code (Baron-Cohen et al., 1993). In the case of printed words, the presence of colors may render them as more distinct. (Although, of course, color cues could lead to memory confusions in certain circumstances for both written and spoken words). Whilst this may explain some of the memory advantage for synaesthetes it is unlikely to be the whole story. For instance, not all memory tests involving color-inducing stimuli have been linked to a memory advantage (e.g., associating digits to spatial positions; Rothen and Meier, 2009), moreover, some visual stimuli that do not induce any synaesthesia are linked to enhanced memory (Rothen et al., 2012). The current set of experiments aims to explore the latter more closely.

Certain visual stimuli that do not induce synaesthesia have been linked to enhanced memory in grapheme-color synaesthetes. Yaro and Ward (2007) found better memory for colors (but normal memory for a complex figure). Pritchard et al. (2013) also showed a memory advantage for color-based associative memory (relative to shape and location based associations). Rothen and Meier (2010) administered a standardized memory test (Wechsler Memory Scale–Revised) to a large group of grapheme-color synaesthetes and found that visual long-term memory was also enhanced and was, in fact, significantly better than verbal long-term memory (which was also enhanced relative to controls). The tests of visual memory in this battery all involved meaningless stimuli (e.g., figures, patterns) both colored and achromatic. Only a few studies have used meaningful visual stimuli (e.g., scenes, objects, faces) and have tended to use small samples of synaesthetes (Gross et al., 2011). As such, evidence for the memory enhancement for visual stimuli presently lacks breadth. The wide variety of testing methods used across different studies (associative memory, recall, recognition) and stimulus properties also make it hard to make direct comparisons across studies and stimuli.

Why do grapheme-color synaesthetes have enhanced visual memory for stimuli not (directly) linked to their synaesthesia? Rothen et al. (2012) suggested that grapheme-color synaesthetes have enhanced visual processing (in certain visual domains) that leads to benefits in both visual perception and visual memory. The latter would extend to certain auditory stimuli (such as spoken words) that are rendered by most grapheme-color synaesthetes as visual objects. This may explain why enhanced memory is not limited to synaesthesia-inducing material. There is evidence that color perception is behaviorally enhanced in grapheme-color synaesthesia (Banissy et al., 2013) but not synaesthesia involving other concurrent modalities such as touch (Banissy et al., 2009). Moreover, grapheme-color synaesthetes show enhanced visual-evoked potential, measured using EEG, to high contrast stimuli, to high spatial frequency gratings, and to chromatic stimuli (Barnett et al., 2008). This has led to the suggestion that the enhanced visual perception is not global but predominantly limited to the parvo-cellular system and/or the ventral visual stream (which depends heavily, but not exclusively, on parvo-cellular inputs). This stream is involved in recognition of visual features (e.g., color), objects, and scenes and is also implicated, in at least some accounts, in long-term storage of this information (Murray et al., 2007). The magno-cellular system is more specialized for motion perception (in addition to low spatial frequency processing and small luminance differences). There is evidence that motion perception is diminished in grapheme-color synaesthesia (Banissy et al., 2013) and that the motion-specialized region, V5/MT, has less gray matter (Banissy et al., 2012). The dorsal ventral stream, involved in processing of egocentric space, depends heavily on the magno-cellular system (e.g., Maunsell, 1987). How these perceptual differences may map onto memory processing in grapheme-color synaesthesia remains to be fully explored, but we speculate that enhanced memory will be more apparent when colors and objects are to be remembered and memory will be unaffected when memory tasks depend heavily on remembering spatial locations, object orientations, and so on.

The present set of experiments makes a significant new contribution to our understanding of memory processing in synaesthesia and addresses some of the limitations of previous research. We consistently use the same methodology for a variety of stimuli, namely a recognition memory paradigm in which a study phase of 30 items is followed by a test phase of 60 items in which participants must discriminate old from new. In Experiment 1, we used four kinds of stimuli presented visually and all achromatic: written words, written non-words, scenes, and fractals. These can be considered as a 2 × 2 design contrasting meaningfulness (words + scenes = meaningful, non-words + fractals = meaningless) and whether the stimulus is language-based (words, non-words) or image-based (scenes, fractals). For both synaesthetes and controls, we would expect better memory for meaningful stimuli because they can be linked to prior knowledge (Ericsson and Kintsch, 1995). If memory enhancement in synaesthesia is related to the presence of “extra” sensations then only the language-based stimuli should be enhanced relative to controls. The enhanced visual processing account would predict that recognition memory for visual stimuli should be generally enhanced (a main effect of group). The same test is administered on a group of lexical-gustatory synaesthetes (Ward and Simner, 2003; Ward et al., 2005) for whom words and, to a lesser extent, non-words (Simner and Haywood, 2009) elicit flavor experiences (e.g., “Philip” tasting of sour oranges). Again, if memory enhancement were linked to extra sensations then we would predict that, for these synaesthetes, it should be specific to words (/non-words). If it is linked to enhanced visual perception then we would not predict any advantage in this sample as we assume (although it is an open question) that the changes in visual perception do not extend to this kind of synaesthesia.

The second study also uses the same recognition memory paradigm but involves scenes contrasted with words. The scenes are manipulated in one of three ways (by changing a color, orientation or object) and the study is loosely based on Pritchard et al. (2013). In that study, participants (synaesthetes and controls) were shown meaningless visual stimuli comprising of a conjunction of shape, color and location (e.g., shape A + color A + location A). In the test phase, they had to distinguish the initial conjunctions from those in which shape had been changed (e.g., shape B + color A + location A), color had been changed (e.g., shape A + color C + location A), or location had been changed (e.g., shape A + color A + location D). Synaesthetes did better, overall, on the test and showed a particularly enhanced ability to reject new items on the basis of color. Experiment 2 uses images of real world scenes in which the color of an object is changed between study and test (e.g., a chair is changed from red to green), or the orientation is changed (mirror reversal), or an object is added/removed from the scene. Our hypothesis is that grapheme-color synaesthetes will have enhanced memory on this test (a main effect of group), and particularly when color is a reliable cue to old/new status (an interaction between group and feature type).

## Experiment 1: recognition memory for words, non-words, scenes and fractals

### Methods

#### Participants

Grapheme-color synaesthetes (*n* = 28) and non-synaesthete control subjects (*n* = 35) were recruited by email. Synaesthetes were tested for consistency using the Eagleman et al. (2007) battery and the cut-off score of 1.43 (from Rothen et al., 2013a). The groups were matched for age and sex, with a mean for synaesthetes of 31.6 years (*SD* = 14.9; 5 male), and 31.1 (*SD* = 12.43; 2 male) years for controls. They were also matched for level of formal education [*X*^2^_(3)_ = 5.29, *p* = 0.15] which was reported on a 4-category scale (postgraduate, undergraduate, to age 18, to age 16). None of them reported any instances of lexical-gustatory synaesthesia.

A sample of lexical-gustatory synaesthetes (*n* = 18) and matched controls (*n* = 18) were additionally tested. The groups were matched for age and sex, with a mean for synaesthetes of 40.9 years (*SD* = 16.5; 4 male), and 36.6 (*SD* = 17.9; 4 male) years for controls. They were also matched for level of formal education [*X*^2^_(3)_ = 3.70, *p* = 0.30] which was reported on a 4-category scale (postgraduate, undergraduate, to age 18, to age 16). Ten of them had taken part in previous published research and 8 were self-referred and not previously assessed in detail. None of them reported any instances of grapheme-color synaesthesia.

Approval was gained for this study through the University of Sussex Life Sciences and Psychology Cluster-based Research Ethics Committee.

#### Design

A 2 × 2 × 2 mixed design was used, with an independent measures factor of group (synaesthete and control), and repeated measures factors of meaningfulness (meaningful and meaningless), and stimulus type (language-based and image-based). The two groups of synaesthetes were compared separately to their respective control groups.

#### Stimuli

All words and nonsense words were four letters long, consisting of one syllable. Nonsense words were used only if they were considered pronounceable in spoken English. Thirty words and 30 nonsense words were selected for use and a further list of 30 words and 30 nonsense words was generated by changing one letter of the original item (e.g., FISH-FIST, SNET-SNEF). The position of the changed letter in the stimulus was varied across the four possible positions (appearing 7 or 8 times in each position). One item from each pair was randomly assigned to be either an old or new item (with the assignment fixed across participants). They varied in both frequency of usage (from 1 to 917; Kucera and Francis, 1967) and imageability. All words and non-words were presented in Calibri font using black upper case letters against a white window, 350 pixels wide by 263 pixels tall.

**Figure 1:**
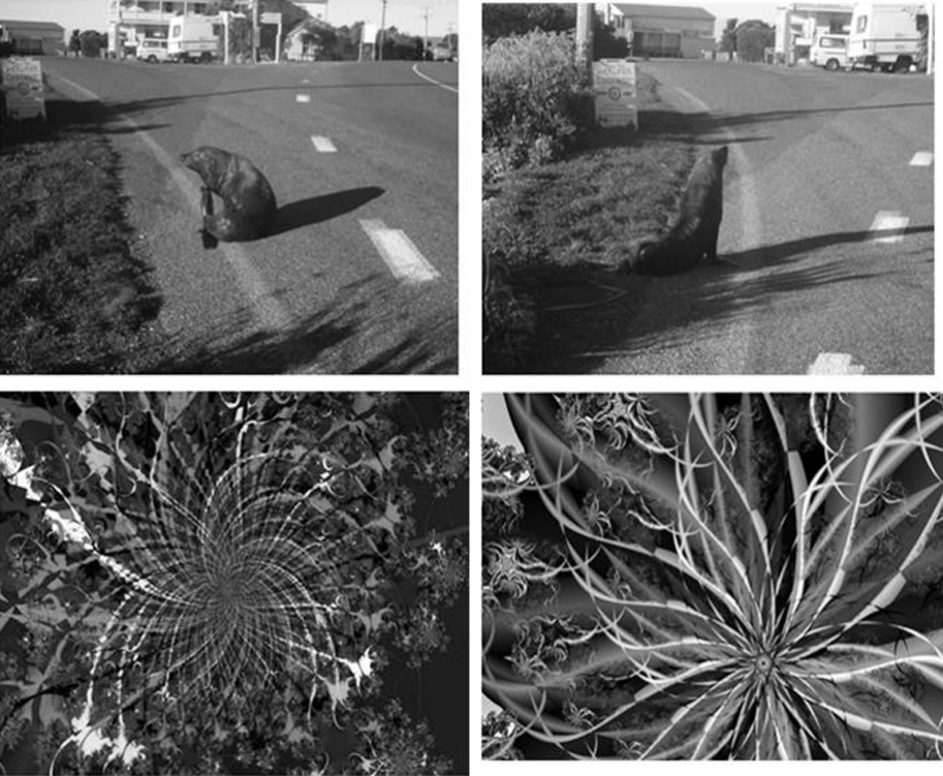
**Examples of pairs of images used as old and new items in Experiment 1 (scenes on top, fractals on the bottom)**.

The scenes consisted of 30 pairs of grayscale images taken by one of the authors (PH). They consisted of rural and urban landscapes containing various objects (e.g., people, animals, vehicles). The pairs were perceptually (and conceptually) similar having been taken from somewhat different viewpoints or with certain objects present or absent. Fractal patterns were downloaded from the internet (Fantastic Fractals, 2011) and were all in grayscale. Similar fractal images were paired together. One item from each pair of scene or fractal images were randomly assigned as either an old or new item (with the assignment fixed across participants). The images were 350 pixels by 263 pixels in size and displayed against a white background. Examples are shown in Figure 1.

#### Procedure

Cake PHP web application software was used to create a program for presenting stimuli to participants over the internet and recording their responses. After providing consent, participants were required to enter an email address so that the software could prevent multiple attempts at completing the test and so that synaesthetes (known to the researchers) could be grouped separately to controls and linked to their synaesthetic consistency score. They were also required to enter their age and sex. After the experiment participants were asked on screen to select their highest level of education achieved.

The main experiment consisted of four sets of study phases followed immediately by test phases (i.e., study-test, study-test, etc.) with the order of stimuli (words, non-words, scenes, fractals) counterbalanced across participants. Before each study phase participants were informed of the stimulus category and were asked to try to remember them for later on. Participants clicked to start the experiment once they had read the instructions. Items within a category were presented in a random order across participants. Each item was presented for 1 second against a plain white background.

After each study phase, participants were presented with another instruction screen, informing them that some items that had been presented and some items that they had not seen before would be displayed, and that they were required to respond “old” or “new” to items respectively. Participants were made aware that new items were similar to those presented before. This phase presented all 60 items from previously studied category and participants made a self-paced mouse-click on the old/new radio buttons. The item remained on screen until participants confirmed their choice, at which point the next item was displayed.

Once all four learning and test phases had been administered participants were asked on screen whether they had seen any colors for each of the stimulus types (with a brief definition of synaesthesia, for the benefit of the controls). Participants were also asked which statement best describes the strategy they had used for each type of stimulus from a list of “Associating with other things,” “Verbalizing the item” or “Visualizing the item.”

### Results

The results for the grapheme-color synaesthetes are considered first, followed by the lexical-gustatory synaesthetes. A value of *p* < 0.05 was used for statistical significance, although we highlight possible non-significant trends (*p* < 0.10) and supplement our analyses with effect size calculations.

**Figure 2:**
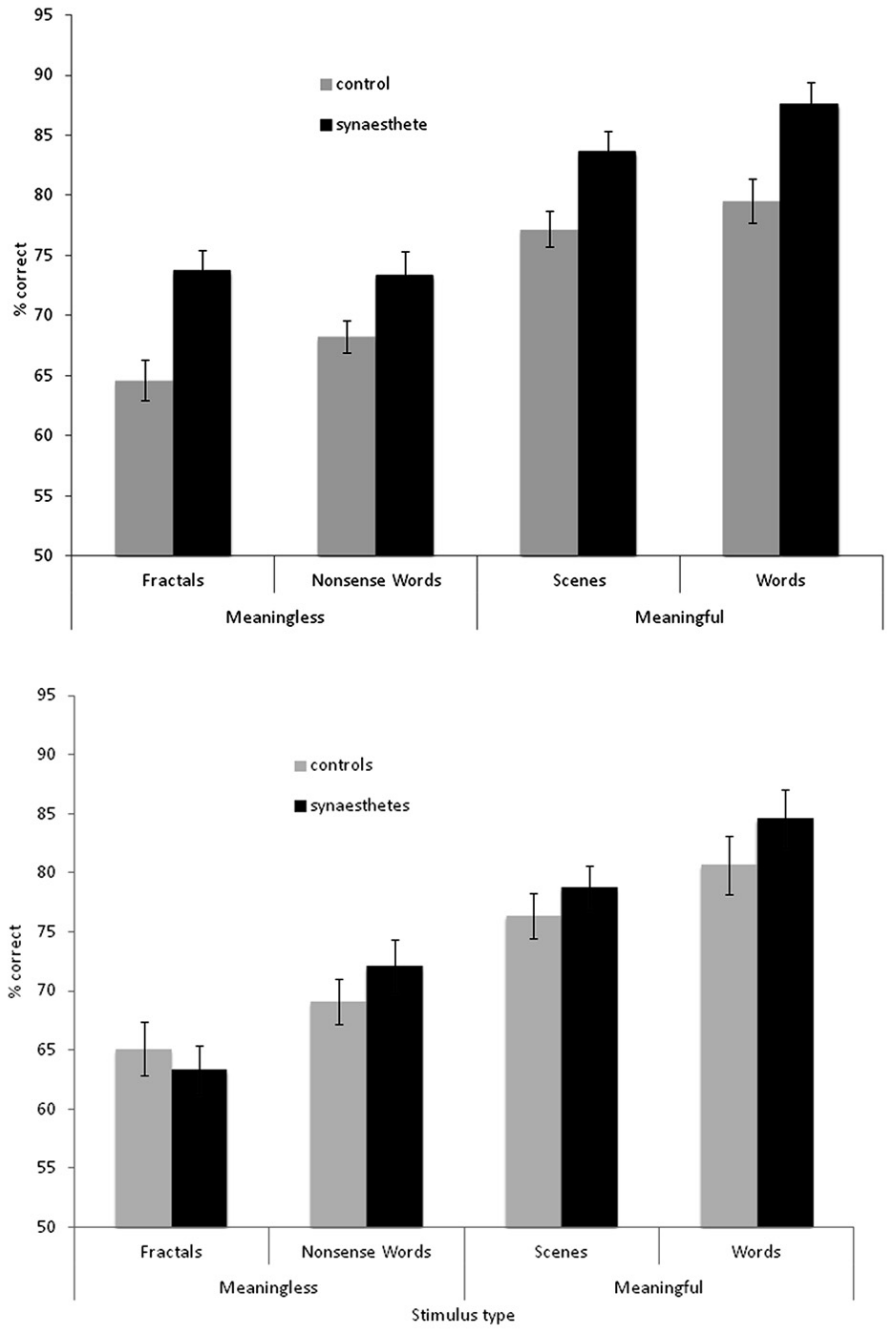
**Recognition memory accuracy (% correct) for synaesthetes and controls for different classes of stimuli.** Top graph: grapheme-color synaesthetes. Bottom graph: lexical-gustatory synaesthetes. Error bars show ±1 SEM.

**Figure 3:**
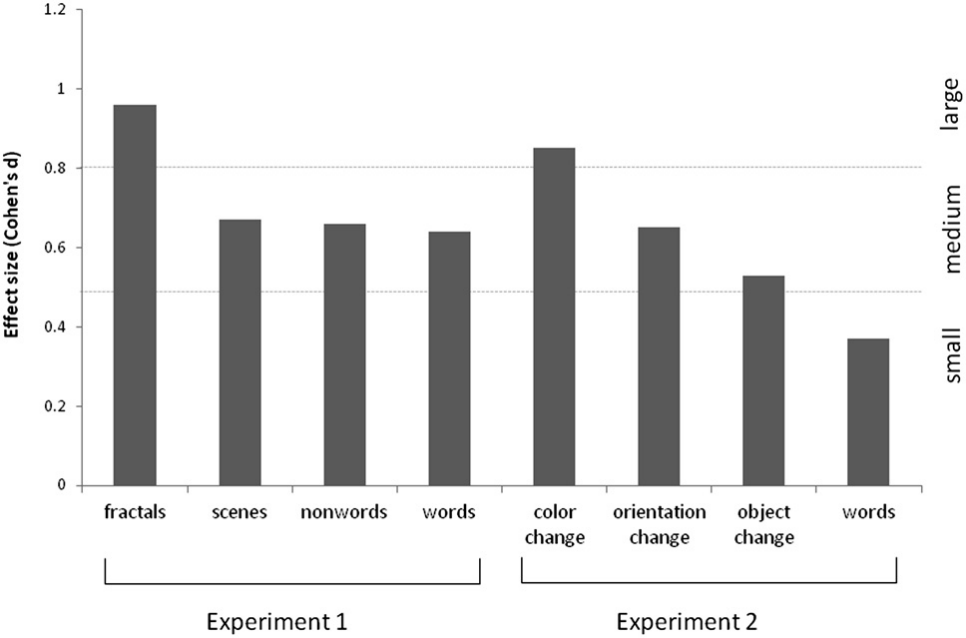
**Effect sizes (Cohen's d) for the various stimuli used in Experiments 1 and 2, for grapheme-color synaesthetes relative to controls.** These are calculated based on the memory discriminability estimates (i.e., d-prime, normalized hits minus false alarms).

The results are summarized in Figure 2 for overall accuracy (percentage correct). A 2 × 2 × 2 mixed ANOVA revealed a main effect of group [*F*_(1, 61)_ = 15.32, *p* < 0.001] with synaesthetes outperforming controls. There were also main effects of meaningfulness [*F*_(1, 61)_ = 207.23, *p* < 0.001] and stimulus type [*F*_(1, 61)_ = 6.99, *p* < 0.01] with meaningful stimuli (words, scenes) being more memorable than meaningless stimuli (non-words, fractals) and linguistic stimuli being more memorable than images. However, neither of these factors interacted with group (*p* > 0.10) suggesting that synaesthetes benefit from these factors similarly to controls. No other interaction was significant but the three-way interaction approached significance [*F*_(1, 61)_ = 3.02, *p* = 0.087]. This is due to a trend for the synaesthetes to do particularly well in recognizing the fractal images. This is shown in Figure 3, considering the effect sizes for the different classes of stimuli. The same overall pattern is reproduced when the hits and false alarms are transformed to d-prime scores with synaesthetes performing better than controls on fractals (synaesthetes: mean = 1.46, *SD* =0.73; controls: mean = 0.84, *SD* = 0.59), non-words (synaesthetes: mean = 1.41, *SD* = 0.73; controls: mean = 1.02, *SD* = 0.44), words (synaesthetes: mean = 2.81, *SD* = 1.19; controls: mean = 2.03, *SD* = 1.25), and scenes (synaesthetes: mean = 2.14, *SD* = 0.77; controls: mean = 1.64, *SD* = 0.74).

In the debriefing questions, the majority of synaesthetes reported noticing colors for words (82%) and non-words (82%) during the experiment. No control did so. However, the controls and synaesthetes generally did not differ in their self-reported strategies for memorizing the different stimuli. This is shown in Table 1. The groups did not differ in their reported strategy for fractals [*X*^2^_(2)_ = 0.09, *p* = 0.76], non-words [*X*^2^_(2)_ = 1.96, *p* = 0.38] or scenes [*X*^2^_(2)_ = 2.53, *p* = 0.28]. That is, the objective benefits in memory performance for these stimuli cannot be attributed to synaesthetes deliberately adopting a different strategy to controls. The reported strategies used for words did, however, differ significantly across groups [*X*^2^_(2)_ = 11.72, *p* = 0.003] with controls tending to rely more heavily on a shallower “verbalizing” strategy (in comparison to the deeper “associating” strategy). This may contribute to the memory advantage for synaesthetes in word recognition and this point is returned to in Experiment 2.

**Table 1:** **Encoding strategies reported by grapheme-color synaesthetes and controls for the various stimuli during the debriefing questionnaire (%)**.

	Words ^**^		**Non-words**		**Fractals**		**Scenes**	
	**Syns**	**Cont**	**Syns**	**Cont**	**Syns**	**Cont**	**Syns**	**Cont**
Associations	57	29	25	40	32	29	21	9
Verbalizing	11	51	36	34	0	0	4	9
Visualizing	32	20	39	26	68	71	75	83

The chi-square statistics are calculated on frequency counts but are shown here as percentages for ease of comparison.

** p < 0.01.

The results for the lexical-gustatory synaesthetes are summarized in Figure 2B. A 2 × 2 × 2 mixed ANOVA revealed no main effect of group [*F*_(1, 34)_ = 1.83, *p* = 0.18]; i.e., these synaesthetes do not show the same memory advantage as documented for the grapheme-color synaesthetes. Most of the synaesthetes reported in the debriefing that they had experienced tastes for the words (94% of participants) and non-words (77%), so we may have predicted a group X verbal/image interaction (with the synaesthetes showing enhanced memory only for verbal stimuli) but this was not found [*F*_(1, 34)_ = 0.07, *p* = 0.799]. Nor were any other interactions significant (all *p*'s > 0.10). As before, there were main effects of meaningfulness [*F*_(1, 34)_ = 80.24, *p* < 0.001] and stimulus type [*F*_(1, 34)_ = 14.22, *p* = 0.001] with meaningful stimuli (words, scenes) being more memorable than meaningless stimuli (non-words, fractals) and linguistic stimuli being more memorable than images. However, a direct comparison between the two groups of synaesthetes (again employing a 2 × 2 × 2 ANOVA) failed to reveal a main effect of group [*F*_(1, 44)_ = 2.81, *p* = 0.101], or any interactions between stimuli and group (all *p* > 0.10). Thus, statistically speaking, it is not possible to reliably distinguish lexical-gustatory synaesthetes from either non-synaesthetes or from grapheme-color synaesthetes. Further studies will need to contrast different types of synaesthesia (e.g., sequence-space) in which sample sizes and demographics are better matched. As an exploratory first step, it is to be noted that the grapheme-color synaesthetes did significantly outperform lexical-gustatory synaesthetes on the fractal stimuli [*t*_(44)_ = 2.26, *p* = 0.029] but not any of the other stimuli (all *p* > 0.10).

### Discussion

Experiment 1 showed that grapheme-color synaesthetes have enhanced recognition memory for visually presented stimuli from a variety of categories. Whilst other studies (Yaro and Ward, 2007; Rothen and Meier, 2010) have contrasted different kinds of stimulus material (words, meaningless figures, etc.) they have tended to use a variety tasks to probe memory (e.g., verbal recall, drawing figures, associating several features/items together) thus making it uncertain whether the effects relate to the stimuli used, the task, or both. By standardizing the task we demonstrate that the memory advantage extends, approximately evenly, across all the stimuli tested. This is inconsistent with the notion that the memory advantage is due specifically to the presence of extra sensations. It is broadly consistent with the notion of enhanced (ventral) visual processing, although we initially predicted that memory for meaningless visual stimuli would be particularly enhanced in grapheme-color synaesthesia because these rely primarily on shallow visual encoding (assumed to be enhanced in these synaesthetes) whereas words/scenes afford opportunities for encoding deeply (we assume the structure of semantic memory to be unaffected by synaesthesia). Nevertheless, fractals showed the largest memory benefit for these synaesthetes which is intriguing given that these images consist of high contrast, high spatial frequency patterns which are known to elicit larger visual evoked potentials in these synaesthetes (Barnett et al., 2008). The memory advantage for these synaesthetes cannot be attributed to the strategy adopted (with the possible exception of words) because the reported strategies tended not to differ between groups. It is impossible to discount the suggestion that the groups differ in ways other than age, sex, and education—for instance in their motivation levels to do well. However, the fact that another group of synaesthetes (with lexical-gustatory synaesthesia) did *not* show a memory advantage, relative to their matched controls, speaks against this (for a similar discussion see Rothen et al., 2013b). It is also noteworthy that the fractal stimuli were associated with the largest group difference (relative to controls) for the grapheme-color synaesthetes but the smallest difference for the lexical-gustatory synaesthetes.

## Experiment 2: recognition memory for scenes differing by color, orientation or object presence

This experiment explores the memory advantage for scenes that was found in Experiment 1. The scenes used in that experiment consisted of pairs of items (one old, one new) that were similar but differed in several ways such as camera angle, the presence/absence of certain objects (e.g., a passing car), as well as the overall pattern of luminance (e.g., presence of shadows). Experiment 2 aims to explore these different visual properties more systematically by using identical images but in which only one aspect of the image is varied at a time (e.g., an object is “airbrushed” out).

### Methods

#### Participants

Grapheme-color synaesthetes (*n* = 33) and non-synaesthete control subjects (*n* = 38) were recruited by email. These were a different sample to that used in Experiment 1. Synaesthetes were tested for consistency using the Eagleman et al. (2007) battery and the cut-off score of 1.43 (from Rothen et al., 2013a). The groups were matched for age and sex, with a mean for synaesthetes of 31.3 years (*SD* = 13.8; 3 male), and 32.9 years (*SD* = 17.61; 5 male) for controls. They were also matched for level of formal education [*X*^2^_(3)_ = 5.33, *p* = 0.15] which was reported on a 4-point categorical scale (postgraduate, undergraduate, to age 18, to age 16). Approval was gained for this study through the University of Sussex Life Sciences and Psychology Cluster-based Research Ethics Committee.

#### Design

The primary interest was in the different kinds of scene manipulations and therefore a 2 × 3 mixed design was used contrasting group (synaesthete and non-synaesthete) and scene manipulation (color change, orientation change, object change). The same word stimuli were included as in Experiment 1, as a fourth condition.

#### Stimuli

**Figure 4:**
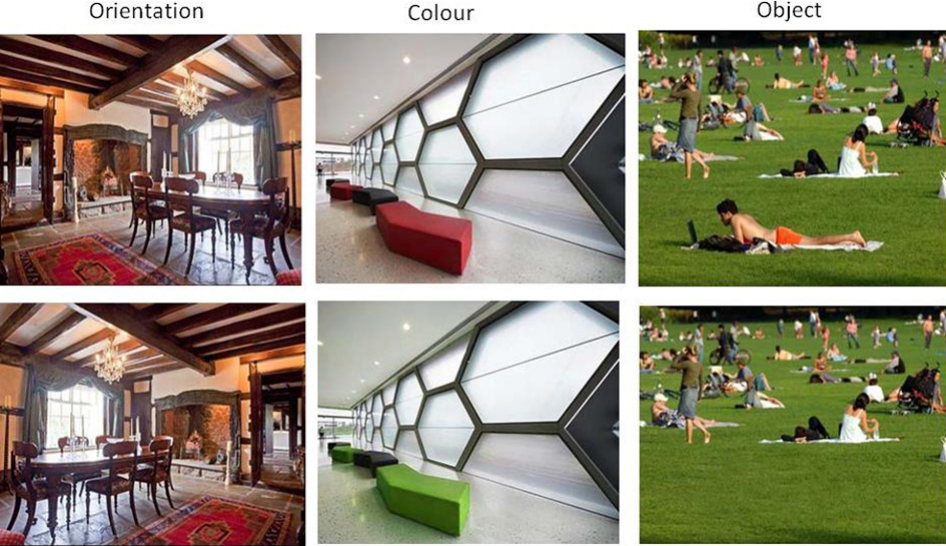
**Examples of pairs of images used as old and new items in Experiment 2**.

The same set of 30 word pairs were used as in Experiment 1. In addition, 3 sets of 30 pairs of outdoor and indoor scene images were created from an initial set of images either taken by an author (AJ) or downloaded from “Google Images” in response to general searches such as “countryside scene” or “picnic.” They were chosen on the basis of being a complex scene but also had a significant feature which could be manipulated without affecting any other aspect of the scene. The original images were manipulated using Adobe Photoshop CS5.1. Thirty pictures were a mirror-image of the original picture, a further 30 were manipulated using an adjustment layer and altering the “hue/saturation” of object(s) in the scene and the final 30 were manipulated using the “clone stamp tool” and “spot healing brush” in order to remove one or more discrete objects from the scene (see Figure 4). Within each pair, one was randomly selected to appear as an old item and one as a new item (i.e., it was not the case the original photos were old items and manipulated items were new). This assignment was fixed across participants.

#### Procedure

The basic procedure was identical to that used in Experiment 1. However, the instructions to participants were modified slightly. At the start of each study phase participants were told whether they would be shown words (1 block) or scenes (3 blocks) to remember but they were not told how the old/new scenes would subsequently differ. Thus, participants had no reason to deliberately try to attend to color, orientation or object presence at encoding. At the start of the *test* phase they were then instructed how the old/new items would differ from each other (e.g., “Click OLD if you have seen it exactly before. Click NEW if it is different in any way. Note the COLORS of some of the objects may have changed”). This was added to ensure that the task was not too difficult and to ensure that participants had properly understood what was required of them. Pilot testing had revealed that participants have a strong tendency to endorse all items as ‘old’ in the absence of this clarification.

### Results

**Figure 5:**
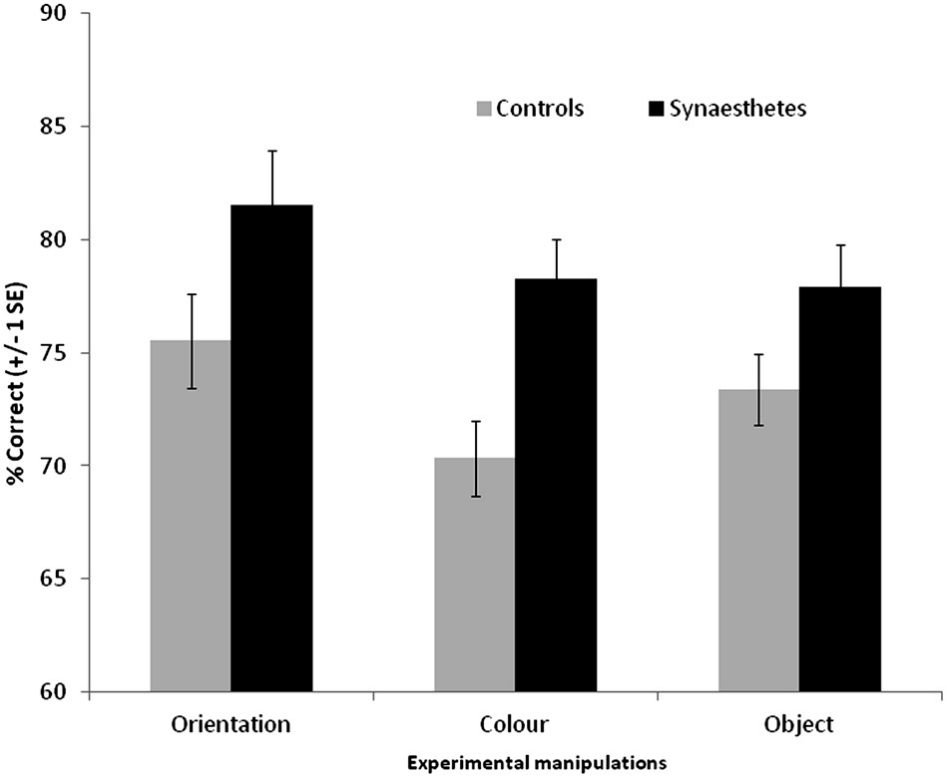
**Recognition memory accuracy (% correct) for grapheme-color synaesthetes and controls for different manipulations of scene images.** Error bars show ±1 SEM.

The results are summarized in Figure 5. For the scenes, a 3 × 2 ANOVA revealed that synaesthetes performed significantly better than controls [main effect of group, *F*_(1, 69)_ = 10.62, *p* = 0.002]. There was a main effect of manipulation [*F*_(2, 138)_ = 3.24, *p* = 0.042] owing to the fact that the orientation changes tended to be easier to detect relative to color [*t*_(70)_ = 2.72, *p* = 0.008; other pairwise comparisons not significant]. Contrary to our hypothesis there was no group X manipulation interaction [*F*_(2, 138)_ = 0.50, *p* = 0.60] suggesting that enhanced memory extends across all conditions. Nevertheless, there was a tendency for the color manipulation to produce the most reliable group difference, as is also evident from the effect sizes displayed in Figure 3.

The same broad pattern is found when the hits and false alarm rates are transformed into d-prime measures. Synaesthetes outperformed controls when orientation was manipulated [means with standard deviation in parentheses: synaesthetes = 2.28 (1.30), controls = 1.58 (0.88)], when color was manipulated [synaesthetes = 1.72 (0.74), controls = 1.15 (0.62)], and when object presence was manipulated [synaesthetes = 1.76 (0.94), controls = 1.35 (0.62)].

The self-reported strategies for memorizing scenes did not differ between synaesthetes and controls [*X*^2^_(2)_ = 0.42, *p* = 0.81]. For synaesthetes, 9% reported “associating to other things,” 9% reported verbalizing, and 82% reported visualizing. For controls, 11% reported associating to other things, 5% reported verbalizing, and 84% reported visualizing. As such, the performance advantage is not attributable to the use of different mnemonic strategies.

**Figure 6:**
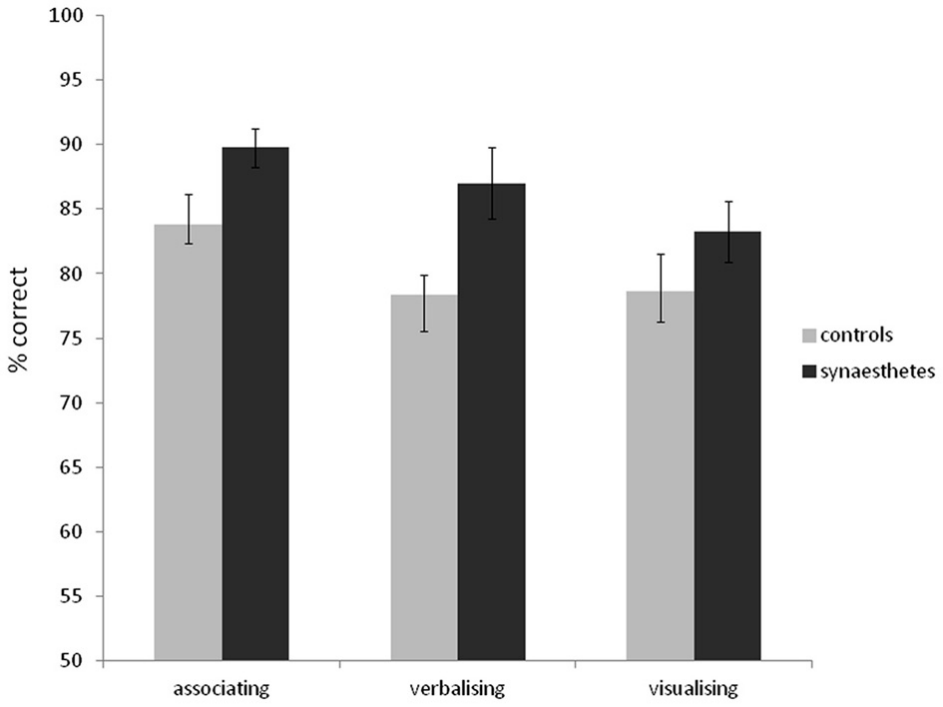
**Recognition memory accuracy (% correct) for grapheme-color synaesthetes and controls for words (collapsing data from Experiments 1 and 2), grouping also according to the strategy used.** Error bars show ±1 SEM.

As in Experiment 1, synaesthetes tended to have better memory for the word stimuli than controls (synaesthetes: mean d-prime = 2.63, *SD* = 1.52; mean % correct = 84.0, *SD* = 10.4; controls: mean d-prime = 2.13, *SD* = 1.26; mean % correct = 80.7, *SD* = 11.8). Unlike in Experiment 1, this did not reach significance [% correct: *t*_(69)_ = 1.25, *p* = 0.21; d-prime: *t*_(69)_ = 1.51, *p* = 0.14]. Most synasthetes reported experiencing colors for the words during the experiment (76%). The self-reported strategies for memorizing words did not differ between synaesthetes and controls [*X*^2^_(2)_ = 0.20, *p* = 0.90]. For synaesthetes, 39% reported associating to other things, 24% reported verbalizing, and 26% reported visualizing. For controls, 34% reported associating to other things, 26% reported verbalizing, and 39% reported visualizing. As such, one of the reasons for the discrepancy between Experiments 1 and 2 may lie in the somewhat different strategies employed (with synaesthetes in Experiment 1 more likely to employ an advantageous “associating” strategy and controls a weaker verbalizing strategy). To examine the effect of strategy use further, we combined the word recognition data across the two experiments and entered strategy as a separate grouping factor. The results are summarized in Figure 6. A 3 × 2 between-subjects ANOVA revealed a main effect of strategy [*F*_(2, 128)_ = 6.68, *p* = 0.002], a main effect of presence of synaesthesia [*F*_(1, 128)_ = 4.47, *p* = 0.036] and no interaction [*F*_(2, 128)_ = 0.48, *p* = 0.62]. In summary, although certain strategies tend to aid the recognition memory for words (notably associating to other things), the memory advantage of synaesthetes is independent of strategy.

Finally, not all of the synaesthetes reported noticing colors for the words (when asked at the end of the study) and the synaesthetes were therefore grouped according to this (collapsing across both experiments). There was no difference in recognition memory ability for words according to whether synaesthetes noticed the colors (mean % correct = 85.8, *SD* = 9.50) or not [mean % correct = 85.3, *SD* = 11.8; *t*_(59)_ = 0.18, *p* = 0.86]. This provides further evidence that the memory enhancement is not directly tied to the deliberate use of synaesthetic color at encoding.

### Discussion

The present study extends that of Experiment 1 by showing that grapheme-color synaesthetes have enhanced visual memory for images of scenes. In Experiment 1 the stimuli were achromatic and in Experiment 2 they were colored, but the memory advantage for grapheme-color synaesthetes were numerically similar for both. That is, the memory advantage for scenes is not dependent on the mere presence of color *per se*. In the present study, the scenes were manipulated in one of three ways: by changing orientation, by changing the color of a surface/object, or by adding/removing an entire object. We predicted, based on other research (Pritchard et al., 2013), that synaesthetes would be particularly good at rejecting distractors on the basis of color. Whilst this condition showed the greatest advantage, the interaction term itself was not significant. It may be that the *spatial distribution* of color (and perhaps other kinds on visual information such as luminance) is crucial, as this was affected by all three manipulations.

Finally, by pooling the data on word recognition memory across the two experiments (which used identical stimuli and testing conditions) we were able to show that the memory advantage for these stimuli, in grapheme-color synaesthesia, is not tied to the adoption of particular strategies and does not depend crucially on whether the synaesthete claimed to have been aware of the colors during the study phase.

## General discussion

The current experiments add to the growing body of evidence that memory is enhanced in grapheme-color synaesthesia and that this enhancement extends to stimuli that are not inducers of synaesthetic color. Most previous studies of visual memory in synaesthesia have used meaningless stimuli, but we extended this to (and directly contrast with) meaningful stimuli notably complex scenes. Based on the theory that enhanced visual memory in grapheme-color synaesthesia is linked to enhanced visual perception (notably of the visual ventral or parvo-cellular system) we predicted that the memory advantage may be greater for meaningless stimuli (fractals)—that tend to be remembered visually—relative to meaningful visual stimuli (scenes) which can be more easily recoded verbally and semantically. This was not found, although there was a trend for the fractal images and color change to be linked to the largest memory benefit.

Within the literature on “superior memory” it has often been found that memory enhancement is strongly domain-specific and directly related to stimuli such as words, digits, and sequences which are more amenable to mnemonic strategies (Wilding and Valentine, 1997). In a difficult test of recognition memory of complex visual patterns (snowflakes) such memory experts typically fare no better than regular controls because their strategies are not helpful in that domain (Maguire et al., 2003). Our prediction is that grapheme-color synaesthetes would show enhanced memory on the snowflake test and, hence, be a convincing example of “true” memory ability that is independent of strategy use or memory training. We have shown that they show an enhancement for visually similar fractal stimuli that are hard to encode semantically and verbally and, indeed, our participants confirm that they tend not to use these strategies. Future studies should attempt to directly impose memorizing strategies (shallow vs. deep encoding) to determine whether the synaesthetic memory advantage is attenuated or increased by these influences (rather than considering strategy *post-hoc*).

With regards to enhanced memory in grapheme-color synaesthesia, it will be as much of a challenge for future research to find memory tests that are *not* linked to enhanced memory as it is to extend and replicate the positive evidence accumulated so far. One starting point may be to consider stimuli in the non-visual domain such as music or voices, as it is possible to devise similar testing protocols to those used in the visual domain. One could also consider non-visual spatial navigation (path integration) or motor learning.

It is also important to determine whether the memory benefits are related to synaesthesia in general or is specifically related to grapheme-color synaesthesia. Our present study failed to find a benefit of having lexical-gustatory synaesthesia. This observation rules out the possibility that enhanced memory in synaesthesia just reflects a motivation to do well (cf. Rothen et al., 2013a,2013b). It is consistent with the notion that enhanced visual perception [assumed to be limited to visual synaesthesia, (Banissy et al., 2009)] leads to enhanced visual memory (Rothen et al., 2012). Another type of visually-based synaesthesia is so-called sequence-space synaesthesia in which sequences such as months, years, and numbers are visualized in a spatial configuration (Eagleman, 2009). It is unclear whether this is linked to enhanced visual perception, but there is some preliminary evidence that they perform well in short-term retention of visual patterns (Simner et al., 2009). These synaesthetes, who visualize years spatially, also perform well at recalling the years in which events occurred and in recalling autobiographical events given a year cue (Simner et al., 2009). This suggests that they are using their synaesthesia as a deliberate memory aid to organize events and is a different kind of explanation to the one proposed here. There is little doubt that, under certain circumstances, synaesthesia can be used as a deliberate memory aid (e.g., Tammet, 2006). However, the current study also demonstrates that grapheme-color synaesthesia can lead to a more general boost in recognition memory for visual stimuli.

### Conflict of interest statement

The authors declare that the research was conducted in the absence of any commercial or financial relationships that could be construed as a potential conflict of interest.
